# Protein restriction during lactation causes transgenerational metabolic dysfunction in adult rat offspring

**DOI:** 10.3389/fnut.2022.1062116

**Published:** 2023-01-10

**Authors:** Rodrigo Vargas, Isabela Peixoto Martins, Camila Cristina Ianoni Matiusso, Raiana Aparecida Casagrande, Camila Benan Zara, Anna Carolina Huppes de Souza, William Pereira Horst, Taina Cristine Sieklicki, Tania Cristina Alexandrino Becker, Naiara Cristina Lucredi, Jurandir Fernando Comar, Ananda Malta, Paulo Cezar de Freitas Mathias

**Affiliations:** ^1^Department of Biotechnology, Genetics, and Cellular Biology, State University of Maringá, Maringá, Brazil; ^2^Health Sciences Center, UniCesumar, Maringá, Brazil; ^3^Department of Morphological Sciences, State University of Maringá, Maringá, Brazil; ^4^Department of Basic Health Sciences, State University of Maringá, Maringá, Brazil; ^5^Department of Biochemistry, State University of Maringá, Maringá, Brazil

**Keywords:** metabolic programming, maternal malnutrition, steatosis, thrifty phenotype hypothesis, metabolism

## Abstract

**Introduction:**

Protein restriction during lactation can induce metabolic dysfunctions and has a huge impact on the offspring’s phenotype later in its life. We tested whether the effects of a maternal low-protein diet (LP) in rats can be transmitted to the F2 generation and increase their vulnerability to dietary insults in adulthood.

**Methods:**

Female Wistar rats (F0) were fed either a low-protein diet (LP; 4% protein) during the first 2 weeks of lactation or a normal-protein diet (NP; 23% protein). The female offspring (F1 generation) were maintained on a standard diet throughout the experiment. Once adulthood was reached, female F1 offspring from both groups (i.e., NP-F1 and LP-F1) were bred to proven males, outside the experiment, to produce the F2 generation. Male F2 offspring from both groups (NP-F2 and LP-F2 groups) received a standard diet until 60 days old, at which point they received either a normal fat (NF; 4.5% fat) or a high fat diet (HF; 35% fat) for 30 days.

**Results:**

At 90 days old, LPNF-F2 offspring had increased lipogenesis and fasting insulinemia compared to NPNF-F2, without alteration in insulin sensitivity. HF diet caused increased gluconeogenesis and displayed glucose intolerance in LPHF-F2 offspring compared to LPNF-F2 offspring. Additionally, the HF diet led to damage to lipid metabolism (such as steatosis grade 3), higher body weight, fat pad stores, and hepatic lipid content.

**Discussion:**

We concluded that an F0 maternal protein restricted diet during lactation can induce a transgenerational effect on glucose and liver metabolism in the F2 generation, making the offspring’s liver more vulnerable to nutritional injury later in life.

## 1. Introduction

A relationship between malnutrition and chronic diseases has been observed worldwide (1). Exponential evidence indicates that perinatal environmental factors, such as maternal malnutrition status, promote long-term effects on the metabolic phenotype of offspring (2, 3); this process is known as metabolic programming. Epidemiological and experimental studies suggest that early life nutritional programming is associated with a higher risk for the development of cardiometabolic syndrome (4–6); an impaired capacity to maintain energy balance is not only limited to exposed individuals but also subsequent generations, even though nutritional conditions are favorable (7).

According to the thrifty phenotype hypothesis, maternal malnutrition provokes metabolic adaptations in offspring that support further development and survival by altered intrauterine growth and an adjusted metabolic phenotype, with a reduced energy demand appropriate for poor nutritional conditions (8–11). However, even an adequate, or excessive postnatal food supply in later life may have negative consequences, mainly on glucose homeostasis (12) and hepatic lipid metabolism (13). This notion is supported by studies on perinatal nutrient restriction during critical periods of development.

Several reports have also focused on the effects of maternal malnutrition. For example, a low-protein diet suggests that the programmed metabolic dysregulation observed in different vulnerability windows may be associated with different biological mechanisms and have a great impact on the phenotype induced in the offspring (7, 14).

Lactation is a window of susceptibility due to the development and maturation of major organs and tissues, which determine the offspring’s metabolic phenotype (2, 15). Thus, exposure to undernutrition during this period can affect metabolism and pancreatic function. Our research group showed that a low-protein diet during lactation increased glycemia, even though offspring displayed higher peripheral insulin sensitivity and lower fasting insulinemia (15). Insulin is a major anabolic hormone involved in hepatic metabolism and dietary protein malnutrition can induce hepatic fat accumulation (16). Maternal low-protein diet effects have also been observed in several studies (17, 18) in the F2 (19, 20) and F3 generations (21, 22), contributing to the early life origin of the risk of chronic diseases.

While the investigation of the transgenerational effects of developmentally programmed traits is widening, very few studies have explored the potential for these traits to be transmitted with post-weaning diets other than adequate controls. In the present study, we assessed the transgenerational transmission of programmed phenotype outcomes on glucose homeostasis and lipidic hepatic metabolism through the maternal lineage and offspring vulnerability to a food insult later in life with a high-fat diet.

## 2. Materials and methods

### 2.1. Ethical approval

All experiments were conducted according to ARRIVE guidelines (23) and Brazilian Association for Animal Experimentation (COBEA) standards. The protocols were approved by the Ethics Committee in Animal Research of the State University of Maringá (n. 5409020520) and performed in the sectional animal facility of the Secretion Cell Biology Laboratory.

### 2.2. Dams

#### 2.2.1. Experimental design and diets

**FIGURE 1:**
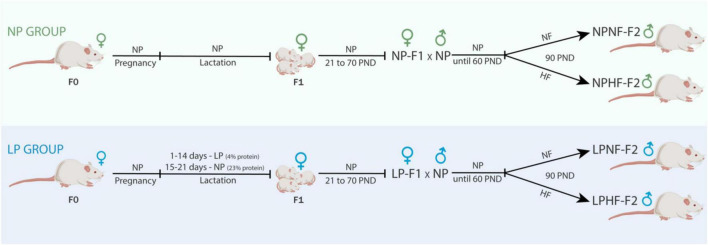
Experimental design. NP, normal-protein diet; LP, low-protein diet; NF, normal-fat diet; HF, high-fat diet; PND, post-natal day.

After 1 week of acclimatization, female and male Wistar rats (70 and 80 days old, respectively) were mated at a ratio of three females to one male. The pregnant females were transferred to individual cages and fed a standard diet. Pregnant F0 females were used to compose the F1 group (Figure 1). At birth, the litter was standardized to eight pups per dam, with a 1:1 sex ratio, and F0 dams were fed either a normal-protein diet (NP; 23% protein; Nuvital; Curitiba/PR, Brazil; *n* = 12) or a low-protein diet (24) (LP; 4% protein; *n* = 12) during the first 14 days of lactation. At postnatal day 21, the F1 female offspring were weaned, housed in groups of four per cage, and fed a standard diet. The F1 male offspring were not evaluated in the present study.

F1 female offspring (*n* = 1 per litter) were kept until 70 days old and mated to proven male rats outside the experiment to compose the NP-F2 and LP-F2 groups. F1 females were fed a standard diet throughout the experimental period. After birth, the litter was standardized to eight pups per dam, in a 1:1 sex ratio. After weaning, only male offspring (NP-F2 and LP-F2) were used in the experiments to avoid estrogen influences.

The experimental procedures were conducted at 90 days old. Throughout the experimental period, the animals were kept under controlled temperature (23 C ± 2°C) and photoperiod (7:00 a.m. to 7:00 p.m., daylight cycle) conditions. Animals received water and food *ad libitum*.

#### 2.2.2. Fasting glycemia and oral glucose tolerance test during pregnancy

Glucose concentration was measured via the glucose oxidase method using a commercial kit (GoldAnalisa; Belo Horizonte, MG, Brazil) (25). On the 18th day of pregnancy (26), after a 6 h fast, blood samples were collected before gavage administration of glucose (1 g/kg of body weight, 0 min, *n* = 6−7) and 15, 30, 45, and 60 min afterward. Glucose response during the test was calculated using the area under the curve (AUC).

#### 2.2.3. Intraperitoneal insulin tolerance test

On the 20th day of pregnancy, an ipITT was performed after a 6 h fast (*n* = 6−7). Dams received an injection of insulin (1 U/kg of body weight) and blood samples were collected as previously reported (27). Subsequently, the rate of glucose tissue uptake or rate constant for plasma glucose disappearance (*K*_*itt*_) was calculated (28).

#### 2.2.4. Biometric parameters and caloric intake during lactation

Body weight (BW) and food intake were measured daily during the suckling phase. Food intake (FI) was calculated as the difference between the amount of remaining diet (D_f_) and the amount presented previously (D_i_): [FI (g) = (D_f_ – D_i_)]. Even though the energy values of the diets were the same, food intake was presented in calories (kcal/100 g of body weight). The AUC for food intake and feeding efficiency [food consumption (g)/body weight (g)] was calculated.

### 2.3. Offspring

#### 2.3.1. Experimental design and diet

At 60 days old, a subset of male offspring from the NP-F2 and LP-F2 groups were fed a normal-fat diet (NF; 7% fat; Nuvital; Curitiba/PR, Brazil) or a high-fat diet (HF; 35% fat) (26) until they were 90 days old. The four experimental groups used were as follows: NPNF-F2, control offspring that were fed an NF diet; LPNF-F2, low-protein offspring that were fed an NF diet; NPHF-F2, control offspring that were fed an HF diet, and LPHF-F2, low-protein offspring that were fed an HF diet (*n* = 8 litter per group).

#### 2.3.2. Body weight gain, caloric intake, feed efficiency, liver weight, and fat pad store measurements

Body weight (BW) and food intake were determined daily from birth to weaning. They were then examined weekly until they reached 90 days old. Food intake was calculated weekly. Considering that the energetic values of the diets were different, food intake was presented in calories. The AUC for food intake and feeding efficiency were calculated. At 90 days old, the rats were anesthetized with thiopental (45 mg/kg of body weight), weighed, decapitated, and laparotomized to remove their liver and retroperitoneal, perigonadal, and mesenteric fat pad stores. The weights of the fat pads and liver were expressed in relation to the BW of each animal (g/100 g of BW).

#### 2.3.3. Intravenous glucose tolerance test

At 90 days old, a batch of animals (*n* = 10−12 rats from 3 to 4 litters per group) was subjected to a surgical procedure to perform ivGTT, as previously described (24). After a 12 h fast, blood samples were collected before the injection of glucose (1 g/kg of body weight, 0 min) and 5, 15, 30, and 45 min afterward. Glucose response during the test was calculated using the AUC.

#### 2.3.4. Intraperitoneal insulin tolerance test

Another batch of animals (*n* = 10−12 rats from 3 to 4 litters per group) was cannulated, and ipITT was performed after a 6 h fast. They received an injection of insulin (1 U/kg of body weight), and blood samples were collected, as previously reported (29). Subsequently, the rate of glucose tissue uptake or the rate constant for plasma glucose disappearance (*K*_*itt*_) was calculated (28).

#### 2.3.5. Blood glucose levels and lipid profile

Glucose concentration was measured by the glucose oxidase method using a commercial kit (GoldAnalisa; Belo Horizonte, MG, Brazil) (25). Triglycerides (TG), total cholesterol, and high-density lipoprotein cholesterol (HDL-C) levels were measured in plasma samples using a colorimetric method and commercial kits (GoldAnalisa; Belo Horizonte, MG, Brazil). Low-density lipoprotein cholesterol (LDL-C) and very-low-density LDL cholesterol (VLDL-C) values were calculated using the Friedewald formula (30).

#### 2.3.6. Hepatic levels of cholesterol and triglycerides

Left-lobe hepatic samples of approximately 100 mg were removed (*n* = 5−10 rats from 5 to 10 litters per group) to determine total lipids using the Folch method (31). The extract was evaporated and then diluted in isopropanol. Cholesterol and TG contents were measured using a commercial kit, in accordance with the manufacturer’s instructions (GoldAnalisa; Belo Horizonte, MG, Brazil).

#### 2.3.7. Pancreas, liver, and retroperitoneal fat histology

Pancreas, liver, and retroperitoneal fat samples (*n* = 5−6 rats from 5 to 6 litters per group) were removed and fixed in 4% paraformaldehyde for 24 h. Subsequently, the samples were dehydrated in an alcohol-increasing series of concentrations. After diaphanization in xylene, the samples were embedded in histological paraffin. Slices of 5-μm thickness were prepared for staining with hematoxylin and eosin (H&E). In the pancreas and fat slices, islets (40 per animal, 40 × magnification) and retroperitoneal fat (20 per animal, 20 × magnification) photomicrographs were randomly acquired using an Olympus DP71 camera coupled to an Olympus BX40 epifluorescence microscope (Olympus, Tokyo, Japan). ImageJ for Windows (Open Source) was used for analysis. Liver slices (30 fields per animal, 20 × magnification) were examined under a light microscope. These values were classified as previously described for the magnitude of steatosis (32). Thus, steatosis was graded as follows: 0 (none to 5% of hepatocytes affected), 1 (>5%−33% affected), 2 (>33%−66% affected), and 3 (>66% affected). The predominant distribution pattern of steatosis was graded as follows: 0 (zone 3), 1 (zone 1), 2 (azonal), or 3 (panacinar).

#### 2.3.8. RNA isolation and real-time quantitative RT-qPCR

Liver samples were collected and stored in liquid nitrogen at −80°C pending total RNA extraction. RNA was isolated from 100 mg frozen tissue using TrizolTM reagent (Thermo Fisher Scientific, Waltham, MA, United States). The RNA concentration was measured using a spectrophotometer at a wavelength of 260 nm (NanoDrop ND 1000 NanoDrop Technologies, Wilmington, DE, United States). cDNA was synthesized using Platus Transcriber RNaseH cDNA First Strand kit (Sinapse Inc., BR), and quantitation of the tissue expression of selected genes was done by quantitative PCR in the Rotor-Gene^®^ Q (Qiagen) with “HOT FirePol^®^ EvaGreen^®^ qPCR Supermix” (Solis BioDyne, EE). The glyceraldehyde 3 phosphate dehydrogenase (GADPH) gene was utilized as a reference gene. The 2^–Δ^ ^CT^ method (33) was used for the relative quantification analysis, and data were expressed as an arbitrary unit (AU). Primers for phosphoenolpyruvate carboxykinase (PEPCK) and fatty acid synthase (FASN) are listed in the Supplementary material.

### 2.4. Statistical analysis

The results were normalized and presented as the mean ± standard error (S.E.M.). Statistical analysis was performed using Student’s *t*-test or two-way analysis of variance, followed by Tukey’s *post hoc* test. *p* < 0.05 was considered statistically significant for the effects of low-protein diet-fed dams (LP), a high-fat diet-fed offspring (HF), or interaction (I) of factors. Analyses were conducted using GraphPad Prism version 6.01 for iOS (GraphPad Software, Inc., San Diego, CA, United States).

## 3. Results

### 3.1. Dams

#### 3.1.1. Body weight, food intake, and glucose homeostasis during pregnancy

**FIGURE 2:**
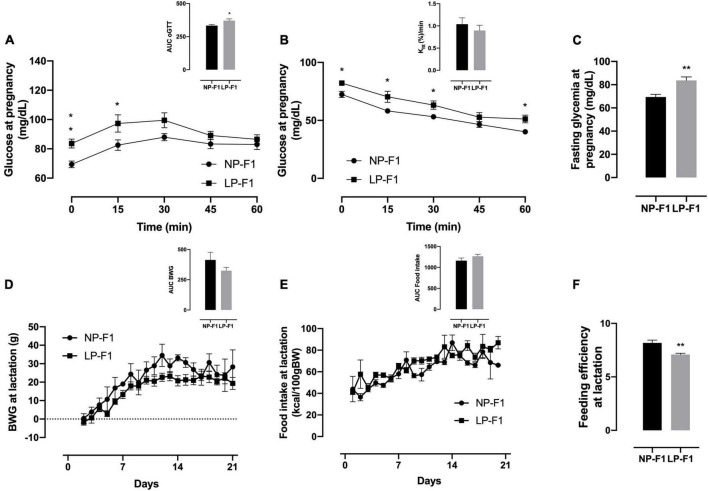
Dams’ biometric parameters and glucose homeostasis analysis. Plasma glucose during oral glucose tolerance test **(A)**, insulin tolerance test and K_itt_ **(B)**, and fasting glycemia **(C)**, during pregnancy; body weight gain **(D)**, food intake **(E)**, and feeding efficiency **(F)** during lactation. The data are expressed as the means ± S.E.M. and were obtained from 6 to 12 dams (from 6 to 12 different litter). The inset represents the AUC. *p < 0.05, **p < 0.01 for Student’s *t*-test. NP-F1, female rats of dams fed a normal-protein diet during lactation; LP-F1, female rats of dams fed a low-protein diet during lactation.

During the oGTT, as observed by the AUC, LP-F1 dams showed mild glucose intolerance (Figure 2D; +10.21%; *p* < 0.05) and fasting hyperglycemia (Figure 2F; +17.02%; *p* < 0.01), without differences in insulin sensitivity, as demonstrated by *K*_itt_ (Figure 2E).

As shown in Figure 2, protein restriction during lactation did not modify BW gain or food intake in dams (Figures 2A, B). However, LP-F1 dams showed a 13.24% decrease in feed efficiency (Figure 2C) during lactation (*p* < 0.01).

### 3.2. Offspring

#### 3.2.1. Biometric parameters and food intake

**FIGURE 3:**
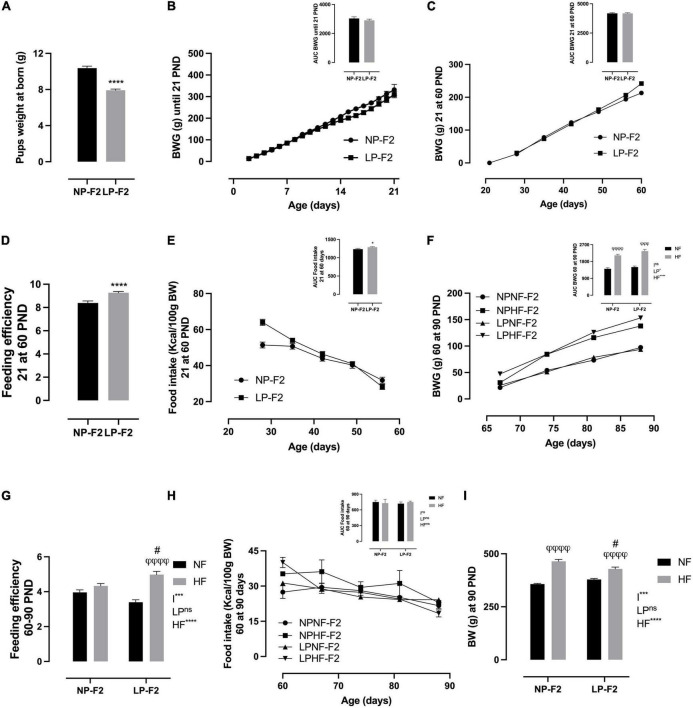
Offspring’s biometric parameters, caloric intake, and feeding efficiency. Litter weight at born **(A)**, body weight gain during lactation **(B)**, body weight gain from 21 to 60 PND **(C)**, feeding efficiency from 21 to 60 PND **(D)**, food intake from 21 to 60 PND **(E)**, body weight gain from 60 to 90 PND **(F)**, feeding efficiency from 60 to 90 PND **(G)**, food intake from 60 to 90 PND **(H)**, and body weight at 90 PND **(I)**. The data are expressed as the means ± S.E.M. and were obtained from 12 to 15 rats of each group (from 4 to 5 different litters). The inset represents the AUC. *p < 0.05, ****p < 0.0001 for Student’s *t*-test. ^#^*p* < 0.05, ^##^*p* < 0.01, ^###^*p* < 0.001, ^####^*p* < 0.0001 to NP-F2 vs. LP-F2 in the same conditions; ^φ^*p* < 0.05, ^φφ^*p* < 0.01, ^φφφ^*p* < 0.001, ^φφφφ^*p* < 0.0001 to NF vs. HF in the same group for the probability based on the Tukey’s *post hoc* analysis. LP, low-protein diet factor; HF, high-fat diet factor; and I, interaction between LP and HF factors. *p < 0.05, ***p < 0.001, ****p < 0.0001, and ns, no significant difference, based on two-way analysis of variance.

Protein restriction caused pup weight at birth to be lower by 23.7% in LP-F2 offspring (Figure 3A; *P* < 0.0001) compared to the NP-F2 offspring. Metabolic programming showed no change in BW gain between offspring groups during the suckling phase (Figure 3B) or until postnatal day 60 (PND) (Figure 3C). However, LP-F2 offspring had increased food intake (Figure 3E; +24.5%; *p* < 0.05) and feed efficiency (Figure 3D; +14.54%; *p* < 0.0001) when compared with the control offspring.

Figure 3F shows that there was no difference in BW gain between LPNF-F2 and NPNF-F2 offspring in adulthood. However, exposure to an HF diet resulted in an increase of 33.68% (*p* < 0.0001) and 36.18% (*p* < 0.001) in body weight gain in NPHF-F2 and LPHF-F2 offspring, respectively. No other differences were observed between the NPHF-F2 and LPHF-F2 offspring. Food intake did not differ between groups (Figure 3H). Additionally, feeding efficiency did not differ between the NPNF-F2 and LPNF-F2 offspring. However, the LPHF-F2 group showed an increase of 31.83% (*p* < 0.0001) and 13.07% (*p* < 0.05) in feeding efficiency compared to the LPNF-F2 and NPHF-F2 groups, respectively (Figure 3G), with interactions between factors.

As shown in Figure 3I, at PND90, there was no difference in bin BW between the NPNF-F2 and LPNF-F2 offspring. The NPHF-F2 group had a 23.14% increase in BW compared with the NPNF-F2 group (*p* < 0.0001). Additionally, the LPHF-F2 group has an 11.58% increase in BW compared to the LPNF-F2 group (*p* < 0.0001). Among the groups that received the HF diet, the LPHF-F2 group had a 7.7% decrease in body weight (*P* < 0.05), with interactions between factors.

#### 3.2.2. Biochemical parameters and lipid profile

As shown in Table 1, fasting glycemia was 15.39% lower in LPNF-F2 offspring than in NPNF-F2 offspring (*p* < 0.05). Offering an HF diet, the NPHF-F2 offspring showed a 13.27% increase in fasting glycemia (*p* < 0.05). However, no difference was observed between LPHF-F2 compared with LPNF-F2 offspring. Nevertheless, the LPHF-F2 offspring showed lower fasting glucose (16.89%) than the NPHF-F2 offspring (*p* < 0.01), with interactions between factors.

**TABLE 1:** Effect of high fat (HF) consumption on biochemical parameters at PND90 of adult F2 offspring from F1 dams programmed by protein restriction on lactation.

Parameters	NPNF-F2	NPHF-F2	LPNF-F2	LPHF-F2	LP	HF	I
Fasting glycemia (mg/dl)	94.7 ± 3.0	109.2 ± 5.5^φ^	80.2 ± 2.7^#^	90.8 ± 3.4^##^	ns	****	**
Fasting insulinemia (ìU/ml)	153.6 ± 18	160.6 ± 5.5	234.9 ± 7.2^###^	253.9 ± 8.8^###^	ns	****	ns
Total cholesterol (mg/dl)	87.6 ± 3.8	123.1 ± 4.0^φ^ ^φ^ ^φ^	88.7 ± 1.4	101.5 ± 3.9^##^	**	*	****
Triglycerides (mg/dl)	66.1 ± 2.4	82.8 ± 6.4	71.6 ± 4.4	90.7 ± 5.5^φ^	ns	ns	**
HDL-C (mg/dl)	51.9 ± 5.1	68.6 ± 2.4^φ^	26.7 ± 2.4^####^	38.9 ± 2.6^####φ^	ns	****	***
LDL-C (mg/dl)	21.2 ± 1.5	30.7 ± 1.6	38.5 ± 1.6^###^	53.4 ± 3.1^####φ^	ns	****	****
VLDL-C (mg/dl)	13.9 ± 0.6	18.1 ± 1.2	14.3 ± 0.9	18.1 ± 1.1^φ^	ns	ns	***

All data are expressed as the mean ± S.E.M. and were obtained from 10 to 12 rats of each group (from 10 to 12 different litters). NPNF-F2, offspring of the dam (F1) born from NP dam (F0) then received NF during adulthood; NPHF-F2, offspring of the dam (F1) born from NP dam (F0) then received HF during adulthood; LPNF-F2, offspring of the dam (F1) born from LP dam (F0) then received NF during adulthood; LPHF-F2, offspring of the dam (F1) born from LP dam (F0) then received HF during adulthood. ^#^*p* < 0.05, ^##^*p* < 0.01, ^###^*p* < 0.001, and ^####^*p* < 0.0001 to NP-F2 vs. LP-F2 in the same conditions; ^φ^*p* < 0.05 and ^φφφ^*p* < 0.001 to NF vs. HF in the same group for the probability based on the Tukey’s post hoc analysis. LP, low-protein diet factor; HF, high-fat diet factor; and I, interaction between LP and HF factors. *p < 0.05, **p < 0.01, ***p < 0.001, ****p < 0.0001, and ns, no significant difference, based on two-way analysis of variance.

Fasting insulinemia was 34.6% higher in the LPNF-F2 offspring compared with NPNF-F2 (*p* < 0.001). The NPHF-F2 and LPHF-F2 offspring showed no differences compared to their counterparts. However, LPHF-F2 displayed an increase of 36.75% in fasting insulinemia compared with NPHF-F2 (*p* < 0.001).

Regarding total cholesterol levels, there was no difference between the LPNF-F2 compared with NPNF-F2 offspring. However, as expected, NPHF-F2 offspring had significantly higher total cholesterol levels than NPNF-F2 offspring (+28%; *p* < 0.001). In contrast, HF diet intake showed no statistical difference in total cholesterol between the LPHF-F2 and LPNF-F2 offspring. Interestingly, LPHF-F2 showed lower cholesterol levels than NPHF-F2 (−17.5%; *p* < 0.01), with interactions between factors.

Triglyceride levels did not differ between the LPNF-F2 and NPNF-F2 groups. Additionally, an HF diet did not increase TG levels in NPHF-F2 offspring compared to NPNF-F2 or LPHF-F2 offspring. However, the LPHF-F2 offspring showed an increase of 21.07% in plasma TG levels compared to the LPNF-F2 offspring (*p* < 0.05), with interactions between factors.

The LPNF-F2 group showed a 48.61% decrease in HDL-C levels compared to the NPNF-F2 group (*p* < 0.0001). As expected, HDL-C levels in the LPHF-F2 group were decreased by 43.29% compared to the NPHF-F2 group (*p* < 0.0001). In addition, an HF diet increased HDL-C levels by 24.41% and 31.50% in the NPHF-F2 and LPHF-F2 groups (*p* < 0.05), respectively, compared to their counterparts, with interactions between factors.

In plasma LDL-C levels, the LPNF-F2 group had levels increased by 44.81% compared with the NPNF-F2 group (*p* < 0.001). Moreover, NPHF-F2 offspring showed a tendency to have increased LDL-C levels compared to NPNF-F2 offspring (*p* = 0.0944). The LPHF-F2 offspring showed an increase of 27.93% and 42.52% compared with LPNF-F2 (*p* < 0.05) and NPHF-F2 offspring (*p* < 0.0001), respectively, with interactions between factors.

Very-low-density LDL cholesterol levels did not differ between the LPNF-F2 and NPNF-F2 offspring. Curiously, the NPHF-F1 offspring were not significantly different from the NPNF-F2 or LPHF-F2 offspring in terms of VLDL-C levels. However, the LPHF-F2 group showed an increase of 21.07% in VLDL-C levels compared to the LPNF-F2 group (*p* < 0.05), with interactions between factors.

#### 3.2.3. Glucose homeostasis during the glucose and insulin tolerance test

**FIGURE 4:**
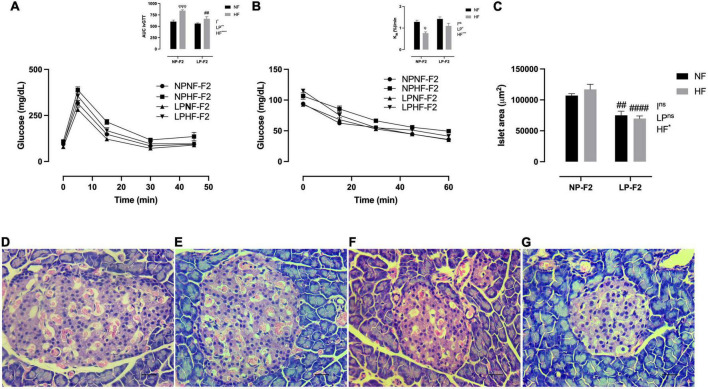
Glucose homeostasis and pancreas morphometry at 90 days old. Plasma glucose during intravenous glucose tolerance test (ivGTT) **(A)**, insulin tolerance test and K_itt_ **(B)**, islet area **(C)**, NPNF-F2 islet **(D)**, NPHF-F2 islet **(E)**, LPNF-F2 islet **(F)**, and LPHF-F2 islet **(G)**. The data are expressed as the means ± S.E.M. and were obtained from 6 to 12 rats of each group (from 3 to 6 different litters). ^##^*p* < 0.01 and ^####^*p* < 0.0001 to NP-F2 vs. LP-F2 in the same conditions; ^φ^*p* < 0.05, ^φφφ^*p* < 0.001 to NF vs. HF in the same group for the probability based on Tukey’s *post hoc* analysis. LP, low-protein diet factor; HF, high-fat diet factor; I, interaction between LP and HF factors. *p < 0.05, **p < 0.01, ***p < 0.001, ****p < 0.0001, and ns, no significant difference, based on two-way analysis of variance.

During the ivGTT, as observed by the AUC, the LPNF-F2 group showed no difference in glycemia or peripheral insulin sensitivity compared with the NPNF-F2 group (Figure 4A), as demonstrated by *K*_itt_ (Figure 4B). As expected, the HF diet resulted in glucose intolerance (Figure 4A; +28.44%; *p* < 0.001) and insulin resistance (Figure 4B; −40.48%; *p* < 0.05) in NPHF-F2 offspring compared with NPNF-F2 offspring. However, no differences were observed between the LP-HF-F2 and LP-NF-F2 offspring in glucose levels and kITT. Although the LPHF-F2 offspring showed significantly lower glucose concentrations during the test than the NPHF-F2 offspring (Figure 4A; −30.36%; *p* < 0.01), no difference was observed in insulin sensitivity (Figure 4B). Glucose tolerance tests showed interactions between the factors.

#### 3.2.4. Pancreatic islet morphometry

Optical analysis showed that pancreatic islet architecture was not altered in the offspring. However, the islet area was lower in the LPNF-F2 (Figure 4F) offspring than in the NPNF-F2 offspring (Figures 4C, D; −29.75%; *p* < 0.01). The LPHF-F2 (Figure 4G) and NPHF-F2 (Figure 4E) offspring showed no difference in islet area compared to their counterparts. However, this parameter was decreased by 40.38% in the LPHF-F2 group compared to that in the NPHF-F2 group (Figure 4C; *P* < 0.0001).

### 3.3. Fat pad store composition and morphometry

**FIGURE 5:**
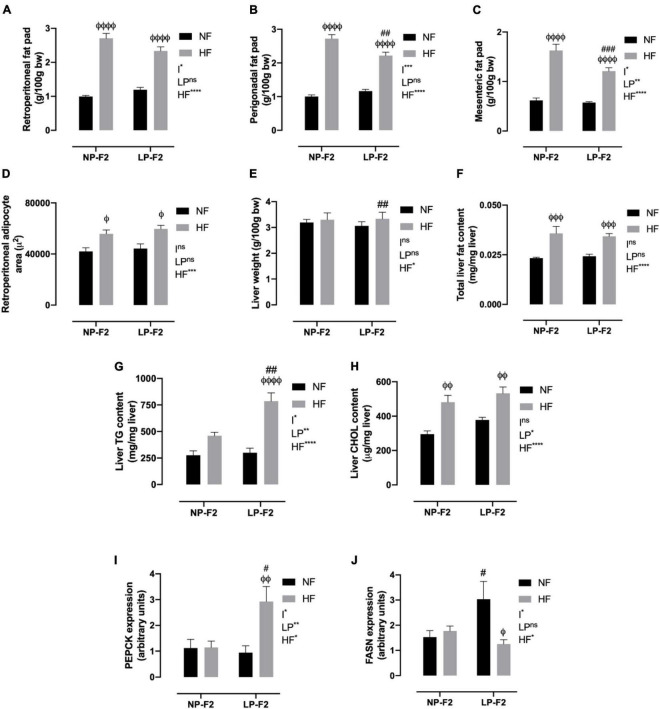
Fat pad store and hepatic profile at 90 days old. Retroperitoneal **(A)**, perigonadal **(B)**, mesenteric fat pad store **(C)**, retroperitoneal adipocyte area **(D)**, liver weight **(E)**, total liver fat content **(F)**, liver triglycerides content **(G)**, liver cholesterol content **(H)**, PEPCK **(I)** and FASN expression **(J)**. The data are expressed as the mean ± S.E.M. and were obtained from 8 to 12 rats of each group (from 3 to 4 different litters). ^#^*p* < 0.05, ^##^*p* < 0.01, ^###^*p* < 0.001, ^####^*p* < 0.0001 to NP-F2 vs. LP-F2 in the same conditions; ^φ^*p* < 0.05, ^φφ^*p* < 0.01, ^φφφ^*p* < 0.001, ^φφφφ^*p* < 0.0001 to NF vs. HF in the same group for the probability based on the Tukey’s *post hoc* analysis. LP, low-protein diet factor; HF, high-fat diet factor; and I, interaction between LP and HF factors. *p < 0.05, **p < 0.01, ***p < 0.001, ****p < 0.0001, and ns, no significant difference, based on two-way analysis of variance.

As shown in Figures 5A(C, no difference was observed in fat pad stores between the LPNF-F2 and NPNF-F2 offspring. As expected, the NPHF-F2 offspring had a higher retroperitoneal (Figure 5A; +63.30%; *p* < 0.001), perigonadal (Figure 5B; +63.19%; *p* < 0.0001), and mesenteric fat pad (Figure 5C; +61.98%; *p* < 0.0001) than the NPNF-F2 offspring. Similarly, the white adipose tissue (WAT) mass was higher in the LPHF-F2 offspring than in the LPNF-F2 offspring, with an increase of 48.84% (*p* < 0.0001), 47.63% (*p* < 0.0001), and 52.64% (*p* < 0.0001) for retroperitoneal, perigonadal, and mesenteric fat pad, respectively. While retroperitoneal fat stores were not different, the LPHF-F2 offspring had lower perigonadal (Figure 5B; −18.48%; *p* < 0.01) and mesenteric fat stores (Figure 5C; 25.68%; *p* < 0.001) compared with the NPHF-F2 offspring, with interactions between factors in all fat pad stores.

Regarding the morphometric analysis of retroperitoneal fat, no differences were observed between the LPNF-F2 and NPNF-F2 offspring. However, the NPHF-F2 and LPHF-F2 groups showed increases of 24.67% and 25.74%, respectively, in the retroperitoneal adipocyte area, compared to their counterparts (Figure 5D; *P* < 0.05). No differences were observed between the LPHF-F2 and NPHF-F2 groups.

### 3.4. Hepatic morphofunction and lipid profile

As shown in Figure 5E, the LPNF-F2 group did not show a difference in liver weight compared with the NPNF-F2 group. Thus, an HF diet did not induce any difference in liver weight in the NP-F2 offspring. However, the LPHF-F2 group showed an increase of 8.18% in liver weight compared with the LPNF-F2 group. No difference was observed in liver weight between the LPHF-F2 and NPHF-F2 offspring.

Regarding total liver fat content, no difference was observed between the NPNF-F2 and LPNF-F2 offspring. However, the HF diet increased liver fat content by 36.11% and 29.41% in the NPHF-F2 and LPHF-F2 offspring, respectively, compared with their counterparts (Figure 5F; *P* < 0.001). No difference was observed in the LPHF-F2 offspring compared to the NPHF-F2 offspring.

Liver TG content was similar between the LPNF-F2 and NPNF-F2 offspring. Curiously, an HF diet did not induce a difference in liver TG content in the NPHF-F2 offspring compared with the NPNF-F2 offspring (Figure 5G). However, liver TG content in the LPHF-F2 offspring increased by 138.17% (*p* < 0.0001) and 158.40% (*p* < 0.01) compared with the LPNF-F2 and NPHF-F2 offspring, respectively, with interactions between factors.

The same pattern of results as for total liver fat content was observed for liver cholesterol content (Figure 5H). No statistical difference was observed between the LPNF-F2 and NPNF-F2 offspring or between the LPHF-F2 and NPHF-F2 offspring. However, a HF diet increased liver cholesterol content by 38.72% and 29.08% (*p* < 0.01) in the NPHF-F2 and LPHF-F2 groups, respectively, compared with their counterparts.

**FIGURE 6:**
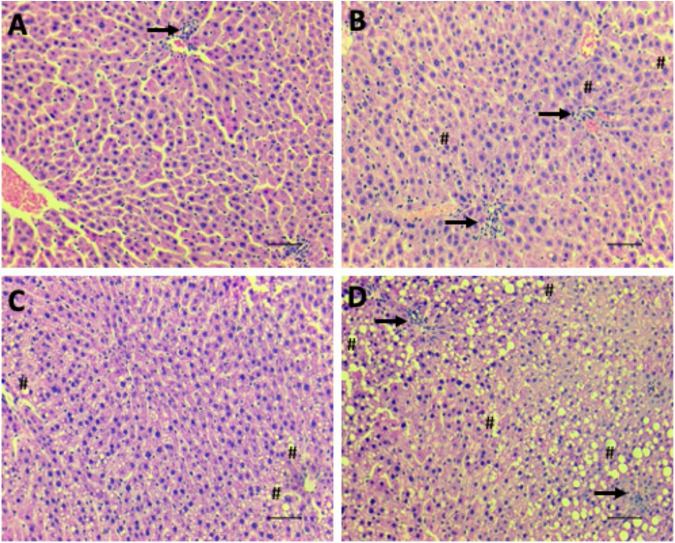
Hepatic steatosis. Representative images of microscopical analyses of steatosis grade 0 (none to 5% of hepatocytes affected) (**A**; NPNF-F2), grade 1 (>5%–33% affected) (**B**; LPNF-F2), grade 2 (>33%–66% affected) (**C**; NPHF-F2) and grade 3 (>66% affected) (**D**; LPHF-F2). Hematoxylin and Eosin-stained sections. Hash, examples of macrovesicular steatosis, in all hepatocytes, fat inclusions displaced nucleus to the periphery; Arrow, cluster of inflammatory cells. Magnification: 200×. Scale bar = 50μm.

The livers of the LPNF-F2 (Figure 6B) and NPNF-F2 (Figure 6A) offspring exhibited a brown-reddish color with no optical evidence of hepatic lipid alteration. Microscopical observation of the hepatocytes showed that they were arranged in rows, delimited by connective tissue containing sinusoids capillaries. As shown in Table 2, NPNF-F2 hepatocytes had a homogeneous cytoplasm without fat vacuoles (score 0; Figure 6A), while the nucleus displayed a central position. LPNF-F2 histopathological analyses showed that 80% of the samples displayed mild steatosis (score 1; Figure 6B) in zone 1.

**TABLE 2:** Effect of HF consumption on hepatic steatosis of adult F2 offspring from dams programmed by protein restriction during lactation.

		% RESPONSES IN CATEGORY				
ITEM		Score	NPNF-F2 (*n* = 5)	NPHF-F2 (*n* = 6)	LPNF-F2 (*n* = 5)	LPHF-F2 (*n* = 5)
**STEATOSIS**						
	<5%	0	100%	0%	20%	0%
	5%−33%	1	0%	0%	80%	0%
	>33%−66%	2	0%	33,33%	0%	20%
	>66%	3	0%	66,67%	0%	80%
**LOCATION**						
	Zone 3	0	0%	0%	0%	0%
	Zone 1	1	0%	50%	100%	0%
	Azonal	2	0%	50%	0%	40%
	Panacinar	3	0%	0%	0%	60%

All data were obtained from 5 to 6 rats of each group (from 5 to 6 different litters). NPNF-F2, offspring of the dam (F1) born from NP dam (F0) then received NF during adulthood; NPHF-F2, offspring of the dam (F1) born from NP dam (F0) then received HF during adulthood; LPNF-F2, offspring of the dam (F1) born from LP dam (F0) then received NF during adulthood; LPHF-F2, offspring of the dam (F1) born from LP dam (F0) then received HF during adulthood.

The livers of the HF-fed offspring presented a yellowish aspect, which macroscopically characterizes hepatic steatosis (Table 2). This was confirmed via histopathological analyses, which showed that the hepatocytes of the NPHF-F2 (Figure 6C) and LPHF-F2 (Figure 6D) offspring exhibited displacement of the nucleus to the cell periphery and the presence of large cytosolic fat vacuoles. These analyses demonstrated that 66.67% of the NPHF-F2 offspring displayed severe macrovesicular steatosis (score 3) distributed in zone 1 (50%) or without a distribution pattern (azonal; 50%). In the LPHF-F2 offspring, a HF diet increased severe macrovesicular steatosis (80%; score 3; Figure 6D) evenly distributed throughout the hepatic tissue (panacinar; 60%).

### 3.5. Liver PEPCK and FASN expression

As shown in Figure 5I, phosphoenolpyruvate carboxykinase (PEPCK) displayed no difference between the LPNF-F2 and NPNF-F2 offspring. Additionally, an HF diet did not induce a difference in PEPCK expression between the NPHF-F2 and NPNF-F2 offspring. However, the LPHF-F2 group showed an increase of 139.10% and 132.17% in PEPCK expression compared with the NPHF-F2 (*p* < 0.05) and LPNF-F2 (*p* < 0.01) groups, respectively, with interactions between factors.

Fatty acid synthase (FASN) was increased by 49.79% in the LPNF-F2 group compared to the NPNF-F2 group (*p* < 0.05) (Figure 5J). Moreover, the NPNF-F2 and NPHF-F2 groups showed no difference in this parameter. FASN expression in the LPHF-F2 group was decreased by 58.75% compared with the LPNF-F2 group (*p* < 0.05). No statistical difference was observed between the LPHF-F2 and NPHF-F2 offspring, with interactions between the factors.

## 4. Discussion

In this study, we evaluated the transgenerational transmission of the programmed phenotype by LP in the first 14 days of lactation through the maternal lineage to adult male F2 offspring and their susceptibility to damage induced by an HF diet later in life. First, we observed that LP alters glucose homeostasis in LPNF-F2 (second generation) offspring, resulting in lower fasting glycemia and islet area. Insulin sensitivity was not altered. However, insulin levels were higher. In addition, we showed lipid hepatic alterations with increased lipogenesis and grade 1 steatosis, verifying the transgenerational effects of the grandmother’s low-protein diet-fed. After an HF diet was offered during adulthood, we show for the first time that the LPHF-F2 offspring are more susceptible to hepatic damage that the NPHF-F2 offspring. They also showed decreased total cholesterol, HDL-C, and fat pad stores. Furthermore, the hepatic tissue is completely compromised by intracellular fat vesicles. These outcomes corroborate Barker’s hypothesis that metabolic programming during critical developmental periods results in altered postnatal metabolism, leaving future generations (such as the F2 generation) more susceptible to diseases (9–11).

The hypothalamus is a well-regulated brain center and an important structure in the control of energy balance (34). The expression of neuropeptides involved in the control of eating behavior is altered in male rats with undernutrition through an increase in the expression of orexigenic hypothalamic peptides, with a concomitant decrease in anorexigenic peptides (35). This results in an increase in caloric intake and a decrease in fat pad stores in adult life (15). In female adult rats, exposure to a low-protein diet during critical periods of development can affect feeding behaviors (36) by causing malformation of the hypothalamus, which remains in adult life (37). However, body weight gain and caloric intake were not altered in F1 dams that were fed a low-protein diet during the suckling phase. In the LP, the removed protein is replaced with carbohydrates to maintain the energy content of the diet. Consumption of these diets can reduce food intake by increasing serotonin production (38).

Several studies have shown that the nutritional status of the dams during critical developmental periods is essential for pups’ normal growth and development (26, 38, 39). Maternal milk is considered a better feeding source for newborns (40), and malnutrition during the suckling phase can negatively affect offspring growth, metabolism, and organ development (15, 26). In addition, offspring health is directly influenced by the intrauterine milieu (41). Here, we show for the first time that LP female offspring F1 displayed fasting hyperglycemia and glucose intolerance during pregnancy. Pregnancy is considered a diabetogenic situation *per se*. Maternal hyperglycemia and diabetes can compromise the food supply and induce adaptations in pancreatic fetal development due to glucose transportation through the placenta (41). Pregnant dams with gestational diabetes mellitus produce offspring with normal or low birth weights (39), which show impaired glucose tolerance during adulthood. This diabetogenic effect can be transmitted to the next generation of individuals (41).

Nevertheless, perinatal undernutrition determines a preference for an HF diet and increases dopaminergic action (42), which indicates the vulnerability of pathways that regulate food intake. However, an increase in HF intake was previously not observed in male offspring LP-F1 (15) and LP-F2 programmed during the suckling phase. An HF diet increased feeding efficiency in the LPHF-F2 offspring compared to the NPHF-F2 offspring, demonstrating catch-up growth. This could indicate a higher risk of obesity and related disorders (43).

An HF diet is directly associated with obesity, dyslipidemia, insulin resistance, and glucose intolerance (15, 38). Indeed, our research group previously showed that these parameters were higher in NPHF-F1 adult rats than in LPHF-F1 rats (15). Here, we show for the first time that LPHF-F2 offspring displayed similar results compared to LPHF-F1 male offspring; thus showing non-genomic phenotype transmission by epigenetic mechanisms from the maternal lineage.

Maternal glucose intolerance can harm lipid metabolism and promote fat accumulation in offspring due to the upregulation of *Insr, Lpl, Pparg*, and *Adipoq* mRNA. Adipocyte hypertrophy is associated with an increase in IL-6 levels, which disrupts insulin signaling (39). The LPHF-F2 group showed high-fat pad gain and an altered lipid profile with a smaller magnitude than the NPHF-F2 group. This transgenerational transmission can contribute to the worldwide pandemic of obesity and type 2 diabetes.

Alterations in lipid profiles have been observed in children with Kwashiorkor syndrome, a deficit in calories and protein. Patients with Kwashiorkor show lower serum TG, and TG accumulation on hepatocytes, due to decreased VLDL-C synthesis. Lower VLDL-C secretion occurs because of a severe protein deficiency. A block in the release of hepatic triglycerides is the major mechanism of fatty liver disease in Kwashiorkor syndrome (44). The LP used in this study had a very low protein content (4%). The restriction of some nutrients in the maternal diet leads to changes in the lipid profile of the offspring (45), such as HDL-C and LDL-C. This is significant as increased LDL-C levels are an important hallmark of cardiovascular disease (46).

A high carbohydrate content in the diet can be associated with decreased HDL-C levels (47). Similarly, LPNF-F2 and LPHF-F2 adult offspring also had lower HDL-C levels, although LP-F1 dams were fed a normal protein diet throughout the experimental period. This profile modification may be due to changes in the expression of the transcription factors that regulate lipolysis and lipogenesis. Suppression of these transcription factors has been shown in rats fed protein restriction during the perinatal period (48), which presented with increased serum TG levels. Triglycerides are the main storage form of energy in adipocytes and hepatocytes in humans and rats. Their release from their stores must be regulated to avoid their toxic potential (49). The liver is an important site for storing excess free fatty acids and is released to control energy homeostasis. TG release occurs in the VLDL-C form by re-esterification at the endoplasmic reticulum with a requirement for apoB and microsomal triglyceride transfer protein (MTP) in the hepatic acinus pericentral zone. Lower VLDL-C secretion leads to the accumulation of TG in hepatocytes, resulting in hepatic steatosis (48).

Hepatic steatosis is characterized by lipid accumulation in hepatocytes, leading to inflammation and the potential progression to liver failure and cirrhosis (50). Even LPHF-F2 offspring had an increase in VLDL-C compared to LPNF-F2 offspring, which is not enough for TG transportation from the liver to the peripheral tissues. Hormonal and dietary factors also affect VLDL-C levels (51). For the first time, we show that protein restriction during the suckling phase had a harmful transgenerational effect on liver tissue when an HF diet was offered. In this study, the LPHF-F2 group displayed higher hepatic TG levels, liver weight, and steatosis grade (score 3) than the NPHF-F2 group.

Fatty acid synthase is one of the major genes responsible for lipid homeostasis and *de novo* lipogenesis and is controlled by hormones and nutritional status. After a meal, blood glucose and insulin levels increase and stimulate *de novo* lipogenesis (52). FASN mRNA expression was higher in the LPNF-F2 offspring, suggesting a compensatory mechanism for energy homeostasis maintenance with low-grade steatosis (score 1). Similarly, a restricted diet during pregnancy increased hepatic FASN mRNA expression in the F0 generation, with a trend in the F1 generation. The increase in FASN mRNA levels correlated with lower HDL-C content in the female progeny (45). Transgenerational studies have shown that LP can affect the hepatic transcriptional profile of thousands of genes until the F3 generation (53). In addition, restricted calorie intake during the preconception or gestational period impairs lipid metabolism, altering FASN mRNA levels in the adult offspring (54). The increase in hepatic TG concentrations during HF feeding is almost entirely driven by uptake and esterification of plasma FFA, without expressive liver contribution. Diets with long-chain fatty acids significantly inhibit *de novo* lipogenesis (55), which could explain low FASN expression in the LPHF-F2 group.

The liver consists of distinct zones with phenotypic heterogeneity, depending on their acinus or lobular localization. Enzymes involved in fatty acid metabolism can exhibit flexibility according to their physiological needs. TG accumulates in hepatocytes starting in the pericentral areas and advancing to the intermediate and periportal areas (56). For the first time, we demonstrated that LPNF-F2 shows steatosis grade 1, with a zone 1 predominant distribution. However, the HF diet increased steatosis to grade 3, with a full distribution of lipids in the liver tissue (panacinar distribution). These results imply that F0 maternal LP could induce a transgenerational effect on liver metabolism and be susceptible to non-alcoholic fatty liver disease in the F2 generation. Liver lipid metabolism is essential to neutralize the impact of FFA-mediated lipotoxicity in peripheral tissues and pancreatic beta cells (56).

The pancreas is the major organ involved in the maintenance of glucose metabolism. Early LP during pregnancy impairs pancreatic beta cell development due to glucose dependence for beta cell maturation, resulting in altered structure and function (14). At the end of gestation, the fetus can control its own glucose levels owing to the adaptation of insulin production and insulin action (26, 41). Studies have shown that poor carbohydrate supply during lactation can change islet structure (37) and stimulate the involution of the pancreas, which is more pronounced in litters from diabetic dams (41). The offspring of hyperglycemic dams display glucose intolerance in adulthood (26). In adulthood, pancreatic mass is normalized (41). In this study, we demonstrated the transgenerational effect of a low-protein diet on glucose homeostasis. The LPNF-F2 group was associated with hypoglycemia and hyperinsulinemia compared to the NPNF-F2 group. Additionally, the LPHF-F2 group showed lower glucose intolerance and hyperinsulinemia than the NPHF-F2 group, insulin sensitivity was not altered, and the islet area was lower. These structural alterations may be compensatory mechanisms to maintain glucose homeostasis early in life (57).

The liver is the only organ that produces and exports glucose. Liver glucose production is dependent on the PEPCK enzyme, one of the main regulators of the gluconeogenic pathway. PEPCK is activated only after birth and catalyzes oxaloacetate decarboxylation to produce phosphoenolpyruvate in the presence of GTP, which is essential for the gluconeogenesis pathway (58). Nutritional status is the primary target of PEPCK regulation. Lipid accumulation is highly associated with higher PEPCK transcription in Zucker genetically fatty rats (59). In LPHF-F2, PEPCK expression increased. This induction may contribute to gluconeogenesis, which can be associated with glucose intolerance and hyperinsulinemia observed in the LPHF-F2 offspring.

## 5. Conclusion

F0 maternal protein-restricted diet during lactation could induce a transgenerational effect on glucose and hepatic metabolism in the F2 generation, making liver offspring more vulnerable to nutritional injury later in life. Some studies show that some results are divergent in the F3 compared to the other generations, due to the direct impact of diet restriction on the development of ovum in F2 females, which did not happen in our study. If dietary restriction changes ovum formation, it could have major translational impacts. However, further studies are required to understand the transgenerational mechanisms of a low-protein diet.

## Data availability statement

The original contributions presented in this study are included in the article/Supplementary material, further inquiries can be directed to the corresponding author.

## Ethics statement

The animal study was reviewed and approved by the Ethics Committee in Animal Research of the State University of Maringá (no. 5409020520).

## Author contributions

RV did the conceptualization, performed the methodology, carried out the formal analysis, investigated the data, and wrote the original draft of the manuscript. IM, CM, RC, CZ, AH, WH, TS, and NL investigated the data. TB and JC carried out the resources and supervised the data. AM and PM carried out the resources, supervised the data, and wrote, reviewed, and edited the manuscript. All authors contributed to the article and approved the submitted version.
