# Supplementary material for: Protein restriction during lactation causes transgenerational metabolic dysfunction in adult rat offspring
Source: Front Nutr. 2023 Jan 10;9:1062116. doi: 10.3389/fnut.2022.1062116 (PMC9872122; doi:10.3389/fnut.2022.1062116)
Supplement: Supplementary file 1 [file Data_Sheet_1.DOCX]

Supplementary material

Sequences of the primers used in PCR

| Gene | Forward | Reverse |
| --- | --- | --- |
| GADPH | TTGTGCAGTGCCAGCCTC | GAGAAGGCAGCCCTGGTAAC |
| PEPCK | GGGGGTGTTTACTGGGAAGG | CGGTTCCTCATCCTGTGGTC |
| FAS | ACCTGTGGAATTCCCGGTTC | ACTCGGAACTGGCGTCAATG |
